# An Air‐Stable Semiconducting Polymer Containing Dithieno[3,2‐*b*:2′,3′‐*d*]arsole

**DOI:** 10.1002/anie.201602491

**Published:** 2016-04-28

**Authors:** Joshua P. Green, Yang Han, Rebecca Kilmurray, Martyn A. McLachlan, Thomas D. Anthopoulos, Martin Heeney

**Affiliations:** ^1^ Department of Chemistry and Centre for Plastic ElectronicsImperial College LondonLondonSW7 2AZUK; ^2^ Department of Physics and Centre for Plastic ElectronicsImperial College LondonUK; ^3^ Department of Materials and Centre for Plastic ElectronicsImperial College LondonUK

**Keywords:** arsenic, conjugated polymers, field-effect transistors, molecular electronics, semiconductors

## Abstract

Arsole‐containing conjugated polymers are a practically unexplored class of materials despite the high interest in their phosphole analogues. Herein we report the synthesis of the first dithieno[3,2‐*b*;2′,3′‐*d*]arsole derivative, and demonstrate that it is stable to ambient oxidation in its +3 oxidation state. A soluble copolymer is obtained by a palladium‐catalyzed Stille polymerization and demonstrated to be a p‐type semiconductor with promising hole mobility, which was evaluated by field‐effect transistor measurements.

Since the first reports of the metallic behavior of conjugated polymers (CPs), the development of new materials has been at the forefront of research in plastic electronics.^1^, ^2^ The enormous scope to tune their optoelectronic characteristics and device performance by the copolymerization of different monomeric units is appealing for many applications.^3^ In recent years, there has been a growing interest in the incorporation of heavier elements—silicon and germanium in Group 14, phosphorus in Group 15, selenium and tellurium in Group 16—as replacements for lighter elements, such as carbon, nitrogen, and sulfur, in CPs.^4^, ^5^, ^6^, ^7^ Their use has been shown to have a significant impact on the polymer band gap and energy levels, as well as the solid‐state packing.^8^ The inclusion of heavy atoms can also facilitate intersystem crossing, leading to the rapid conversion of singlet excitons into triplets, and can lead to solid‐state phosphorescence.^9^, ^10^

Among the CPs containing Group 15 elements, those incorporating a dithienometallole (Figure 1) have garnered much attention. For example, dithieno[3,2‐*b*:2′,3′‐*d*]pyrrole (X=N) and dithieno[3,2‐*b*:2′,3′‐*d*]phosphole (X=P, DTP) have seen application in all major areas of plastic electronics.^11^, ^12^, ^13^ The interest in DTPs partially arises from their high electron affinity, which results from the σ–π hyperconjugation exhibited by the phosphole ring, whereby the σ* orbital of the exocyclic P−C bond is able to interact with the π* orbitals of the fused heterocycle.^14^ Furthermore, modification of the P lone pair, through reactions with either Lewis acids or oxidizing agents, can further tune the energy levels.^15^ However, as phosphole derivatives are prone to uncontrolled oxidation in ambient atmosphere, the phosphole oxide is often deliberately formed to prevent uncontrolled aging.^16^ As such, the properties of the unoxidized phospholes are rarely reported.

**Figure 1:**
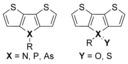
Structures of pnictogen‐containing dithienometalloles.

Inspired by the fascinating properties of DTP, we were interested in preparing the heavier analogue containing a bridging arsenic atom. Of particular interest was the fact that arsenic‐containing compounds are typically more difficult to oxidize to the +5 oxidation state than their P analogues.^17^, ^18^, ^19^ This is related to the poor shielding of the filled 3d orbitals in As, such that the s electrons are relatively tightly bound compared to those in the corresponding phosphorus‐based compounds. Therefore, arsole‐containing polymers may be intrinsically more resistant to ambient oxidation than their phosphole equivalents, allowing the properties of the heterocycle in the +3 oxidation state to be explored. As far as we are aware, there have been no reports of the use of arsole‐containing materials in organic electronics and only limited examples of conjugated polymers containing arsenic in the backbone, mostly related to poly(vinylene arsine)s.^20^, ^21^, ^22^

Herein, we present the first synthesis of a dithienoarsole (DTAs) monomer and demonstrate that it is stable to oxidation under ambient conditions. Significantly, we show that it is possible to directly polymerize the DTAs derivative in its +3 oxidation state by a palladium‐catalyzed Stille polymerization. To the best of our knowledge, the equivalent Pd‐catalyzed polymerizations with DTP derivatives have only been reported for the +5 oxidation state (commonly DTP oxides).^16^ To demonstrate the utility of the DTAs building block, we synthesized the vinylene copolymer PDTAsV (see Scheme 1), in which the vinylene comonomer plays an important role in allowing the backbone to planarize, thereby leading to a promising organic field effect transistor (OFET) performance.^23^, ^24^

One issue in planning a synthetic route to DTAs is the toxicity of many arsenic‐containing precursors. We therefore utilized dichlorophenylarsine as the arsenic building block as its synthesis from a readily available precursor in one step is well established.^25^ Although it is a strong lachrymator and vesicant, it has a history of safe use in synthetic chemistry.^26^ However, one potential drawback of dichlorophenylarsine is that the resulting 4‐phenyldithieno[3,2‐*b*;2′,3′‐*d*]arsole (DTAs) would only have a phenyl substituent as the solubilizing sidechain, possibly resulting in polymers with limited solubility. As such, we incorporated additional dodecyl sidechains into the peripheral positions of the DTAs to ensure good polymer solubility and processability.

The synthesis of the DTAs monomer is shown in Scheme 1. Compound **1** 27 was desilylated with TBAF, and the resulting compound **2** was reacted with *n*‐BuLi at −78 °C, followed by quenching of the resulting dianion with dichlorophenylarsine to afford dithienoarsole **5** in 57 % yield. Attempts to brominate **5** under electrophilic conditions (NBS) resulted in some competing oxidization of the arsole ring to the arsole oxide. However, **5** could be cleanly brominated in 60 % yield by dilithiation with LDA at low temperature, followed by the addition of carbon tetrabromide as a non‐oxidizing halogen source.

**Scheme 1:**
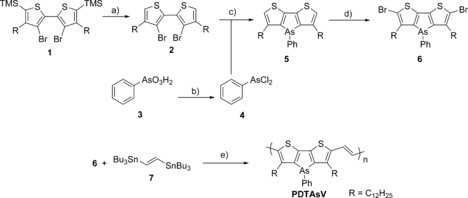
Synthesis and polymerization of the dithienoarsole monomer. Reaction conditions: a) tetrabutylammonium fluoride (TBAF), THF, 0 °C; b) HCl, SO_2_, 55 °C; c) *n*‐BuLi (2.2 equiv), −78 °C→RT; d) lithium diisopropylamide (LDA), −78 to −30 to −78 °C, CBr_4_; e) Pd(PPh_3_)_4_, chlorobenzene, microwave reactor.

With monomer **6** in hand, we investigated its oxidation to the arsole oxide, initially using hydrogen peroxide, which gives almost quantitative yields when used to oxidize dithienophosphole.^13^ However, the reaction was found to be low‐yielding when DTAs was used, with around 60 % of the unoxidized material being recovered. Using *meta*‐chloroperoxybenzoic acid as a stronger oxidant resulted in an improved yield of the oxide of **6**, although starting material still remained even with excess *m*‐CPBA. The oxidation was readily monitored by ^1^H NMR spectroscopy, with the phenyl resonances shifting downfield and splitting upon oxidation (Figure 2). In agreement with the reduced reactivity of the monomer compared to DTP, samples of **6** stored under ambient conditions displayed no signs of oxidation after six months. The reluctance of the DTAs to oxidize is in agreement with the poor shielding ability of the filled 3d orbitals, and is in stark contrast to the DTP analogue. We note that 2,5‐diarylarsoles were very recently be reported to be more air‐stable than their phosphole analogues.^28^

**Figure 2:**
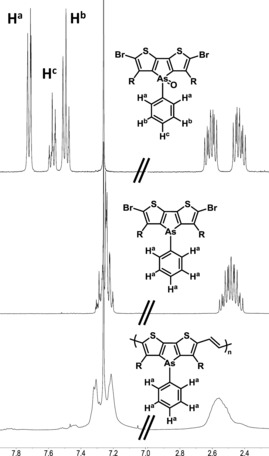
Comparison of the ^1^H NMR spectra of **6** (middle), the oxide of **6** (top), and PDTAsV (bottom) between 7.10–7.90 ppm.

Stille polymerization of **6** with *trans*‐1,2‐bis(tributylstannyl)ethene using catalytic Pd(PPh_3_)_4_ was performed in a microwave reactor to yield PDTAsV (Scheme 1). Gratifyingly, the presence of the arsenic atom, a possible site for binding to the Pd catalyst, did not seem to have any detrimental effects. This finding is in line with the weaker donicity of triphenylarsine versus triphenylphosphine as ligands for palladium.^29^ After precipitation and work‐up, PDTAsV was obtained as a dark blue polymer of reasonable molecular weight (*M*_n_=12.3 kDa, Ð=1.9 by size exclusion chromatography).

The chemical structure of the polymer was confirmed by a combination of elemental analysis and ^1^H NMR spectroscopy. Although the ^1^H NMR resonances were significantly broadened in comparison to the monomer material, no signals attributable to uncontrolled oxidation to the arsole oxide were observed (Figure 2). Together with the matching elemental analysis, these results provide good evidence for the stability of the polymer under the polymerization conditions.

The absorption spectra of PDTAsV in chlorobenzene solution and as a thin film are shown in Figure 3 a. In solution, PDTAsV shows an absorption maximum (*λ*_max_) at 616 nm with a pronounced shoulder (*λ*_sh_) at 652 nm. Upon film formation, the *λ*_max_ value of PDTAsV was red‐shifted to 638 nm, whereas *λ*_sh_ was shifted to 682 nm. The red shift is suggestive of enhanced molecular ordering compared to the solution state. Further evidence of solid‐state ordering was obtained by X‐ray diffraction (XRD) of drop‐cast films before and after thermal annealing for 30 min at 200 °C (Figure 3 b). The as‐cast sample displayed a broad, low‐intensity peak with a *d*‐spacing of 21.0 Å. Upon annealing, this peak became sharper and significantly enhanced in intensity, which is indicative of an increase in molecular ordering. The *d*‐spacing increased to 22.6 Å, which is very similar to the value observed for other bridged bithiophene–vinylene co‐polymers with dodecyl sidechains, and attributed to the distance between conjugated backbones in a lamellar‐type arrangement.^30^

**Figure 3:**
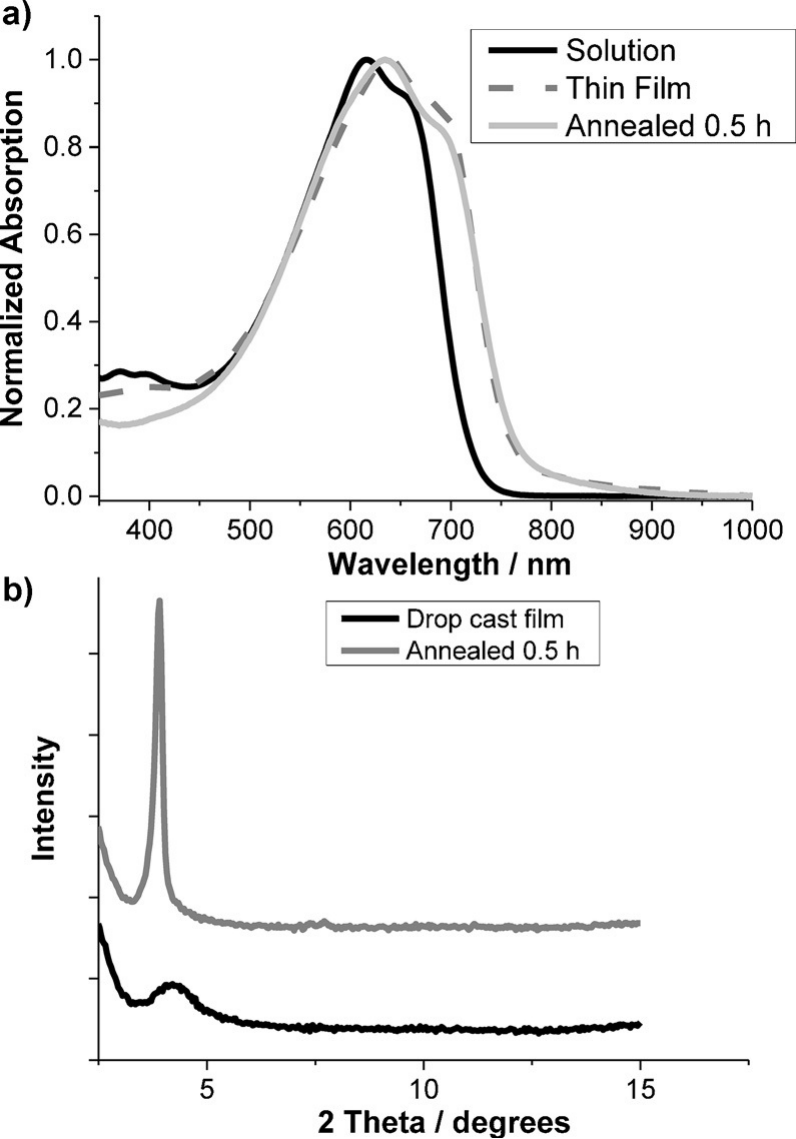
a) UV/Vis absorption spectra of PDTAsV in chlorobenzene solution (black), as a thin film on a glass slide (dashed), and after annealing at 200 °C for 30 min (gray). b) XRD patterns of PDTAsV films drop‐cast onto silicon wafers, as‐cast (black) and after annealing at 200 °C for 30 min (gray). Traces offset for clarity.

The XRD data and UV/Vis spectra suggest that the polymer is able to planarize and crystallize in the solid state. To further probe the monomer and polymer geometries, DFT calculations were performed at the B3LYP level of theory with the 6‐311G(d,p) basis set. Initially considering the monomer, the calculations predicted the bridged bithiophene to be perfectly co‐planar whilst the phenyl group attached to the bridging arsenic atom is twisted orthogonally to the fused‐ring structure, and is almost perpendicular to the molecular plane (Figure 4 a). The predicted frontier molecular orbitals (Supporting Information, Figure S11) show that the highest occupied molecular orbital (HOMO) has a nodal plane through the arsenic atom whereas the lowest unoccupied molecular orbital (LUMO) has some contribution from the σ* orbital of the As−Ph bond.

**Figure 4:**
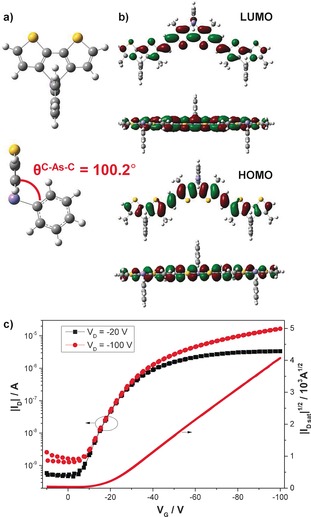
a) Predicted geometry of the dithienoarsole monomer and b) frontier molecular orbitals of PDTAsV, as calculated by DFT [B3LYP, 6‐311G(d,p)]. c) Representative transfer characteristics measured with a top‐gate/bottom‐contact architecture in nitrogen atmosphere at room temperature.

Moving to the polymeric system, minimum energy models of the trimer predict a highly planar backbone with twists of <1° between the DTAs and adjacent double bonds (Figure 4 b). The insertion of the double bond between the head‐to‐head dodecyl sidechains clearly reduces any undesirable torsional twisting between the DTAs units. Furthermore, the side‐on view clearly shows that the phenyl rings protrude perpendicularly to the conjugated backbone. Note that in the minimum‐energy conformer shown, the phenyl groups are predicted to alternate above and below the plane of the backbone, but there is no energy difference between trimers with alternative arrangements, and we therefore expect a random arrangement in the actual polymer. It is also worth noting that the barrier to inversion of the phenyl group in arsole has been predicted to be large owing to the limited p character of the lone pair,^31^ and we would expect similar in DTAs. Finally, the frontier molecular orbital calculations (Figure 4 b) show that whereas both the HOMO and LUMO are extensively delocalized over the polymer backbone, only the LUMO has a significant contribution on the As bridge, similar to the monomer.

Finally, we tested the electrical performance of PDTAsV in a top‐gate/bottom‐contact transistor geometry. The PDTAsV was annealed at 200 °C for 30 min prior to deposition of the gate dielectric (Cytop™). As can be seen in Figure 4 c, the devices displayed p‐type behavior, with negligible hysteresis between the forward and reverse sweeps. The polymer exhibited promising average hole mobilities, measured in saturation, of 0.08 cm^2^ V^−1^ s^−1^, with a current on/off ratio of approximately 10^4^. Importantly, the hole mobility was relatively independent of the gate voltage, and did not display the peak observed at low gate voltage for some materials (Figure S12). To the best of our knowledge, this is the only report of an arsole‐containing compound exhibiting macroscopic charge transport in an electronic device. The promising performance suggests that the presence of the arsenic bridging group with the phenyl substituent does not inhibit the close overlap of the conjugated backbones needed for charge transport.

In conclusion, we have reported the synthesis of the first dithienoarsole heterocycle and shown that unlike the analogous dithienophosphole, it is stable to oxidation in the presence of ambient air. We incorporated the dithienoarsole into a soluble conjugated polymer backbone, which can aggregate and crystallize in the solid state and demonstrates promising charge‐carrier mobility in OFET devices. We believe that these results show that dithienoarsole is an interesting building block for organic electronics. In particular, the opportunity to tune the oxidation state of the bridging arsenic atom may offer interesting opportunities for the utilization of these materials in sensing applications.

## Supporting information

As a service to our authors and readers, this journal provides supporting information supplied by the authors. Such materials are peer reviewed and may be re‐organized for online delivery, but are not copy‐edited or typeset. Technical support issues arising from supporting information (other than missing files) should be addressed to the authors.

Supplementary
