# Supplementary material for: An Air‐Stable Semiconducting Polymer Containing Dithieno[3,2‐b:2′,3′‐d]arsole
Source: Angew Chem Int Ed Engl. 2016 Apr 28;55(25):7148–51. doi: 10.1002/anie.201602491 (PMC4999038; doi:10.1002/anie.201602491)
Supplement: Supplementary file 1 — Supplementary [file ANIE-55-7148-s001.pdf]

Angewandte Chemie
Eine Zeitschrift der Gesellschaft Deutscher Chemiker

Supporting Information

An Air-Stable Semiconducting Polymer Containing Dithieno[3,2-*b*:2′,3′-*d*]arsole

*Joshua P. Green, Yang Han, Rebecca Kilmurray, Martyn A. McLachlan, Thomas D. Anthopoulos, and Martin Heeney**

anie_201602491_sm_miscellaneous_information.pdf

Supporting Information

Contents

1. Experimental details
2. NMR spectra of novel compounds
3. Heated UV of **PDTAsV**
4. Frontier molecular orbitals of **DTAs** monomer
5. OFET output characteristics
6. References

1. Experimental details

General techniques

3,3'-Dibromo-4,4'-didodecyl-[2,2'-bithiophene]-5,5'-bis(trimethylsilane) (**1**) was synthesized by a previously published route.[1] Reagents were purchased from Sigma Aldrich and VWR and used with no additional purification, and all reactions were performed using oven-dried glassware under argon using standard Schlenk techniques. Thin layer chromatography (TLC) was performed on silica plates (Merck Kieselgel 60 F_{254} aluminum sheets) and visualized using UV light (254 and 365 nm). Column chromatograph was performed on silica gel (Merck Kieselgel 60 230–400 mesh). ^{1}H and ^{13}C NMR spectra were recorded using Bruker AV-400 spectrometers (^{1}H = 400 MHz, ^{13}C = 101 MHz) at 298 K. Microwave experiments were carried out in a Biotage Initiator (v. 2.3). UV-visible spectroscopy was carried out using a UV-1800 Shimadzu UV-vis spectrometer for both solution and thin-film samples. Thin films of the polymer were prepared by spin coating a 5 mg ml^{-1} solution of **PDTAsV** in chlorobenzene at 1000 rpm for 60 s. Molecular weight (M_n, M_w, PDI) was measured using an Agilent Technologies 1200 series GPC at 80 °C, using chlorobenzene as an eluent with two PL mixed B columns in series. The column was calibrated using narrow polydispersity polystyrene standards. X-ray diffraction (XRD) measurements were carried out with a PANalytical X'Pert-pro MRD diffractometer equipped with a nickel-filtered Cu Kα source (1.54 Å) and X'Celerator detector, using current I = 40 mA and accelerating voltage V = 40 kV. Samples were prepared by drop casting onto silicon substrates (100) and annealing was carried out for 30 min at 200 °C under argon. Samples were rotated at 60 rpm during measurements. Cyclic voltammetry (CV) was performed in a standard three-electrode setup with polymer film on FTO as the working electrode, a Pt mesh as the counter electrode and an Ag wire as the reference electrode. The working electrode was prepared by spin coating a 10 mg/ml solution of **PDTAsV** in chlorobenzene onto FTO-coated glass (supplied by Sigma Aldrich, ~7 Ω/sq) at 1000 rpm for 60 s. Voltammograms were measured using a 0.1 M tetrabutylammonium hexafluorophosphate ((TBA)PF_6) solution in acetonitrile as an electrolyte, at a scanning rate of 0.05 V/s. Redox potentials were calibrated against a standard ferrocene/ferrocenium (FOC) system, assuming the energy level of FOC to be 4.8 eV below vacuum. Geometries and frontier molecular orbitals were calculated using density functional theory at the B3LYP level of theory with the 6311G(d,p) basis set, using the GAUSSIAN09 software package.[2]

OFET device fabrication

Top gate/bottom contact devices were fabricated on glass substrates using Au (60 nm) source-drain electrodes and CYTOP dielectric. Au electrodes were treated with pentafluorobenzene thiol (PFBT) SAM to increase the work function. Polymer was dissolved in chlorobenzene at a concentration of 10 mg/ ml, and spun cast at 2000 rpm for 60 s. The obtained polymer film was annealed at 200 °C for 30 min before spin coating of CYTOP dielectric. The channel width and length of the transistors were 1000 µm and 40 µm, respectively.

Synthesis of 3,3'-dibromo-4,4'-didodecyl-[2,2'-bithiophene] (2)

To a solution of **1** (11.11 g, 13.8 mmol) in anhydrous THF (120 ml) at 0 °C was added a solution of TBAF (1M in THF, 32 ml, 2.3 eq) in three portions, and the resulting solution was stirred for 5 min. Hexane (250 ml) was added, followed by a further 10 min of stirring. The resulting precipitate was removed by filtration through a short silica plug, and the solvent removed under reduced pressure to afford **2** (8.76 g, 13.3 mmol, 96%) as a yellow solid (m.p. 59-60 °C). ^{1}H NMR (400 MHz, $CDCl_3$) δ 7.10 (s, 2H), 2.69 – 2.52 (m, 4H), 1.66 (m, 4H), 1.45 – 1.22 (m, 36H), 0.88 (t, *J* = 6.8 Hz, 6H). ^{13}C NMR

(101 MHz, $CDCl_3$) δ 142.44, 121.87 (2C), 115.62, 32.08, 30.95, 29.84, 29.81 (2C), 29.75, 29.59, 29.51 (2C), 29.18, 22.85, 14.28. HRMS (EI^+) calculated for $C_{32}H_{51}S_2Br_2$ [M^+-H], 657.1799; found, 657.1808.

Synthesis of dichlorophenylarsine[3] (4)

Caution: *$PhAsCl_2$ is highly toxic and a vesicant, and as such should be handled with extreme care. Proper safety equipment should be used at all times, and reactions should be carried out in a well-ventilated fumehood. Treat any spillages and all glassware with dilute bleach to prevent accidental contamination and injury.*

Phenylarsonic acid (**3**) (4.99 g, 24.7 mmol) was placed in a flask, and HCl (37%, 50 ml) was added, followed by stirring until **3** was completely dissolved. Trace I_2 was added, and the reaction was heated to 55 °C while SO_2 was bubbled through the reaction for 6 h. The reaction was then left undisturbed overnight to allow phase separation to occur, after which the dense bottom layer (containing product) was removed, and the top layer extracted with $CHCl_3$. The combined organic fractions and initial bottom layer were concentrated in vacuo, after which the product was purified by vacuum distillation (~71 °C at 0.5 mbar) to yield the red liquid, **4** (4.94 g, 22.2 mmol, 89%). ^1H NMR (400 MHz, $CDCl_3$) δ 7.91 – 7.85 (m, 2H), 7.57 – 7.51 (m, 3H). ^{13}C NMR (101 MHz, $CDCl_3$) δ 145.43, 132.28, 130.01 (2C), 129.34 (2C). HRMS (EI^+) calculated for $C_6H_5Cl_2As$, 221.8984; found, 221.8980.

Synthesis of 3,5-didodecyl-4-phenyldithieno[3,2-*b*;2',3'-*d*]arsole (5)

To a degassed solution of **2** (2.00 g, 3.0 mmol) in anhydrous THF (120 ml) at -78 °C as added *n*-BuLi (2.5M in hexane, 3.64 ml, 9.1 mmol, 3 eq) dropwise over 10 min. After stirring for 1 h at 78 °C, **4** (0.41 ml, 3.0 mmol, 1 eq) was added slowly, and the reaction was allowed to warm to RT overnight. The reaction was quenched with methanol (10 ml), stirred for 30 min and the solvent was removed under reduced pressure. The resulting low melting point solid was dissolved in hexane, filtered through silica (eluent: hexane) to remove salts and then purified by column chromatography over silica (eluent: hexane), to afford **5** as a low melting point, off-white waxy solid (1.09 g, 1.7 mmol, 57%). ^1H NMR (400 MHz, Chloroform-d) δ 7.30 – 7.26 (m, 2H), 7.25 – 7.17 (m, 3H), 6.80 (s, 2H), 2.55 – 2.49 (m, 4H), 1.51 – 1.41 (m, 4H), 1.35 – 1.07 (m, 40H), 0.88 (t, J = 6.8 Hz, 6H). ^{13}C NMR (101 MHz, $CDCl_3$) δ 149.12 (2C), 143.93 (2C), 143.09, 136.99, 133.09 (2C), 129.07 (2C), 128.83 (2C), 120.14 (2C), 32.09 (2C), 30.86 (2C), 29.86 - 29.81 (overlapping signals, 8C), 29.66 (2C), 29.52 (2C), 29.47 (2C), 29.35 (2C), 22.85 (2C), 14.28 (2C). HRMS (EI^+) calculated for $C_{38}H_{57}S_2As$, 652.3118; found, 652.3118.

Synthesis of 2,6-dibromo-3,5-didodecyl-4-phenyldithieno[3,2-*b*;2',3'-*d*]arsole (6)

Fresh LDA was prepared by treating a solution of diisopropylamine (0.29 ml, 2.1 mmol, 3.1 eq) in anhydrous THF (10 ml) at -78 °C with *n*-BuLi (2.5M in hexane, 0.80 ml, 2.0 mmol, 3 eq), followed by stirring for 10 min. This was then added dropwise over 10 min to a degassed solution of **5** (0.43 g, 0.66 mmol) in anhydrous THF (25 ml) at -78 °C. The resulting mixture was allowed to warm to -30 °C over 30 min, and then cooled back to -78 °C. A freshly prepared solution of CBr_4 (1M in THF, 2.65 ml, 2.65 mmol), 3.0 mmol) was cooled to -78 °C and added dropwise via syringe. The mixture was allowed to warm to RT overnight, and was then concentrated under reduced pressure. The solid was dissolved in CH_2Cl_2 and filtered through basic alumina (eluent: CH_2Cl_2). The solvent was removed under reduced pressure and the residue recrystallized (ethanol) to give the product, **6**, as a pale yellow solid (0.32 g, 0.39 mmol, 60%, m.p. 65-66 °C). ^1H NMR (400 MHz, $CDCl_3$) δ 7.33 – 7.18 (m, 5H), 2.57 – 2.40 (m, 4H), 1.45 – 0.99 (m, 40H), 0.93 – 0.84 (m, 6H). ^{13}C NMR (101 MHz, $CDCl_3$) δ 147.91

(2C), 142.70 (2C), 141.40, 136.03, 133.07 (2C), 129.63 (2C), 129.08 (2C), 108.57 (2C), 32.09 (2C), 29.99 (2C), 29.80 (6C), 29.58 (2C), 29.52 (2C), 29.40 (2C), 29.32 (2C), 29.14 (2C), 22.86 (2C), 14.28 (2C). MS (MALDI) m/z = 810.2 (M^+). Anal. calcd. for $C_{38}H_{55}AsBr_2S_2$: C, 56.30; H, 6.84. Found: C, 56.28; H, 6.80.

Synthesis of 2,6-dibromo-3,5-didodecyl-4-phenyldithieno[3,2-*b*;2',3'-*d*]arsole-4-oxide (6-oxide)

To a solution of **6** (50 mg, 0.06 mmol) in dichloromethane (2 ml) was added *m*-CPBA (17 mg, 0.10 mmol, 1.67 eq) in one portion. The reaction was stirred for 5 minutes, after which the solvent was removed in vacuo and the residue purified by column chromatography on neutral alumina (eluent: 20% $CHCl_3$ in hexane). This yielded **6-oxide** as a yellow solid (41 mg, 0.05 mmol, 83%). ^{1}H NMR (400 MHz, $CDCl_3$) δ 7.74 – 7.71 (m, 2H), 7.61 – 7.55 (m, 1H), 7.52 – 7.46 (m, 2H), 2.61 (ddd, *J* = 14.1, 9.3, 6.2 Hz, 2H), 2.43 (ddd, *J* = 14.0, 9.4, 6.4 Hz, 2H), 1.39 – 1.01 (m, 40H), 0.88 (t, *J* = 6.8 Hz, 6H). ^{13}C NMR (101 MHz, $CDCl_3$) δ 142.98 (2C), 142.83 (2C), 135.46, 132.97 (2C), 131.62, 130.17 (2C), 129.89 (2C), 111.56 (2C), 32.08 (2C), 29.77 (6C), 29.64 (2C), 29.55 (2C), 29.51 (2C), 29.30 (2C), 29.27 (2C), 29.22 (2C), 22.85 (2C), 14.28 (2C). HRMS (ES) calculated for $C_{38}H_{56}S_2AsBr_2O$ (for [M^++H], based on 100% abundance of ^{79}Br), 825.1355; found, 825.1393.

Stille polymerization of 6 with *trans*-1,2-bis(tributylstannyl)ethene

An oven-dried high-pressure microwave tube was charged with **6** (82.2 mg, 0.101 mmol), *trans*-1,2-bis(tributylstannyl)ethene (61.4 mg, 0.101 mmol), and $Pd(PPh_3)_4$ (2.3 mg, 0.002 mmol). The tube was sealed with a septum and flushed with Ar, after which degassed chlorobenzene was added (0.5 ml). The mixture was thoroughly degassed with Ar, after which the inlet was removed. The vial was then placed in a microwave reactor and heated as follows: 4 min at 100 °C, 120 °C, and 140 °C, 20 min at 160 °C, and 120 min at 180 °C. After cooling to room temperature, the resulting polymer was precipitated into methanol and filtered into a Soxhlet thimble. The polymer was extracted (Soxhlet) with methanol, acetone, and hexane in that order. The hexane fraction was concentrated under reduced pressure and the resulting polymer was dissolved in chloroform, warmed to 50 °C and stirred in the presence of diethylammonium diethyldithiocarbamate[4] (ca. 20 mg) for 1 hr. The solution was concentrated in vacuo, dissolved in a minimal amount of hot chlorobenzene and the polymer collected by re-precipitation into methanol followed by vacuum filtration. After drying under vacuum, polymer **PDTAs-V** was collected (54 mg, 79%). GPC (chlorobenzene): M_n 12,300 g/mol, M_w 23,700 g/mol, Đ 1.92. ^{1}H NMR (500 MHz, 323 K, $CDCl_3$) δ 7.31 (broad, 2H), 7.21 (broad, 3H), 6.92 (broad, 2H), 2.56 (broad, 4H), 1.26 (broad, 40H), 0.87 (broad, 6H). Anal. Calcd. For $(C_{40}H_{57}AsS_2)_n$: C, 70.97; H, 8.49. Found: C, 70.91; H, 8.42.

2. NMR spectra of novel compounds

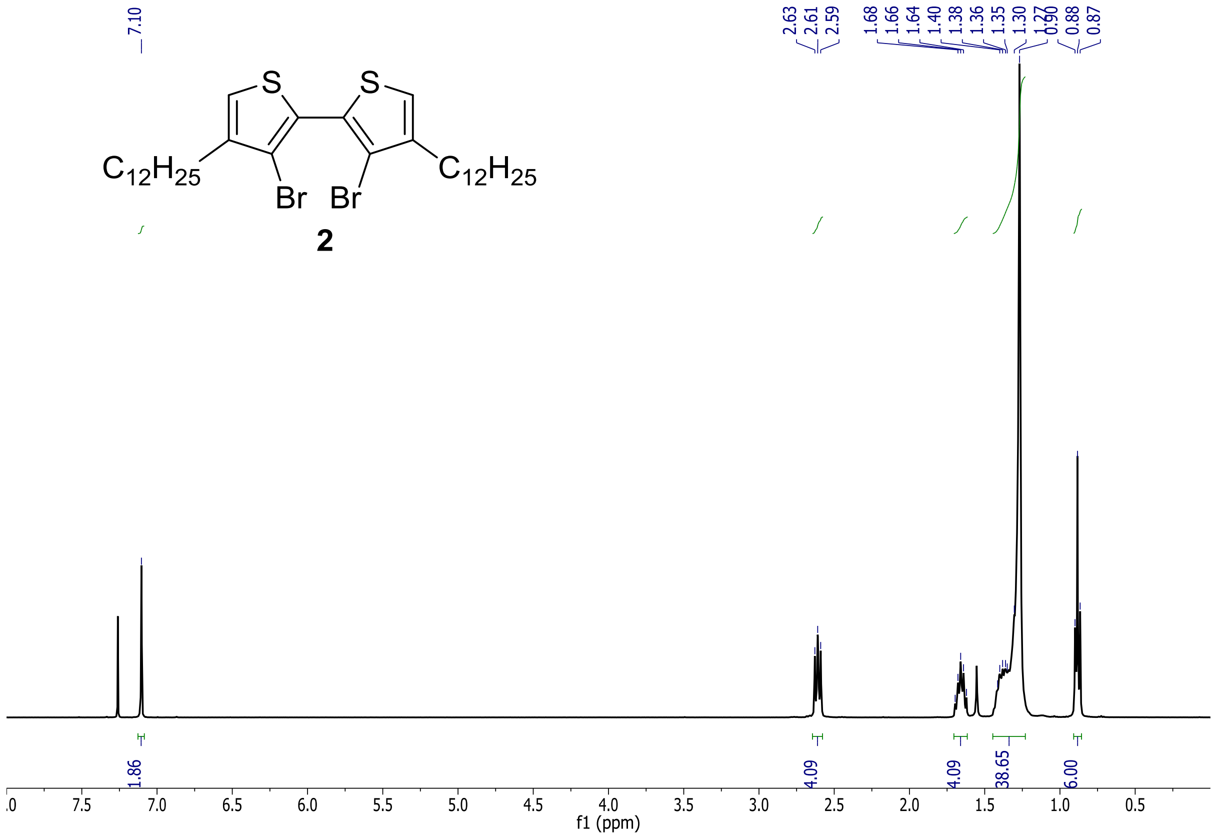

Figure S1: ^{1}H NMR spectrum of **2** (400 MHz, $CDCl_3$).

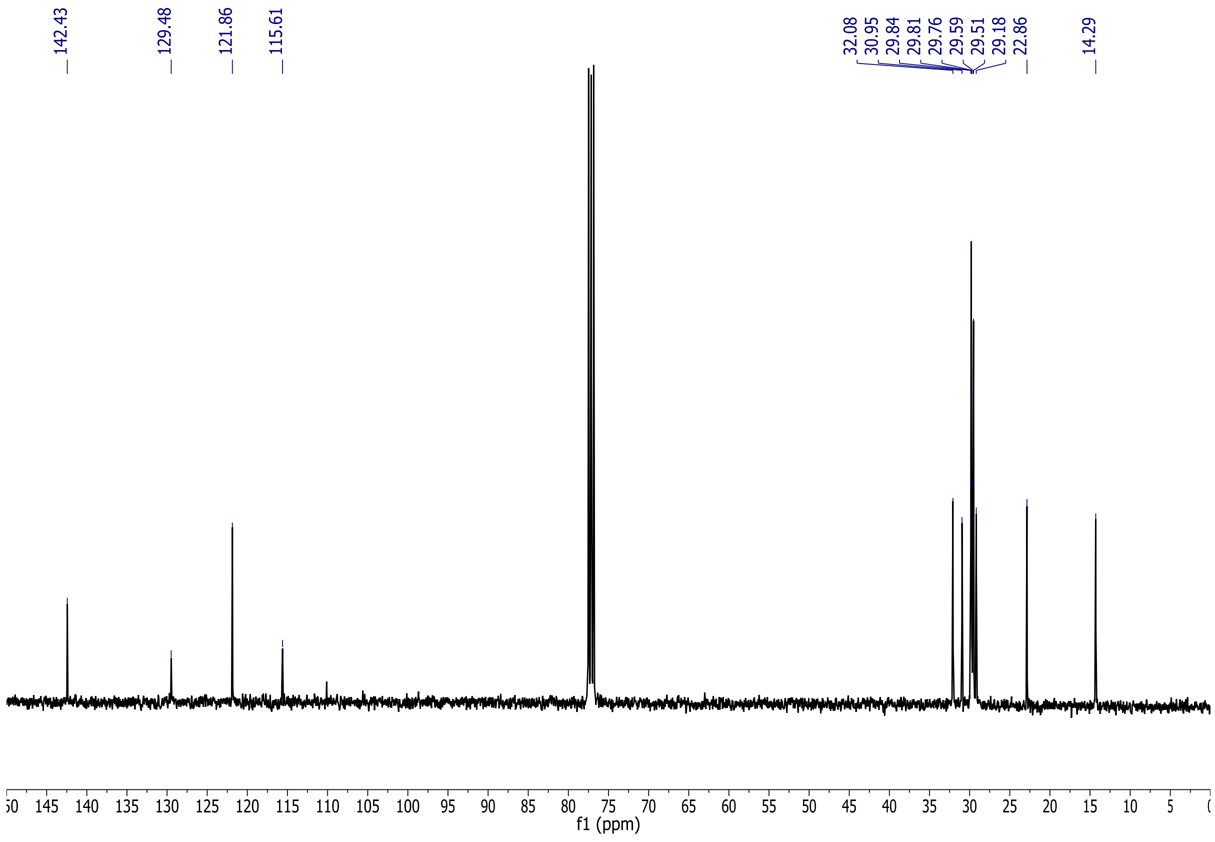

Figure S2: ^{13}C NMR spectrum of **2** (101 MHz, $CDCl_3$).

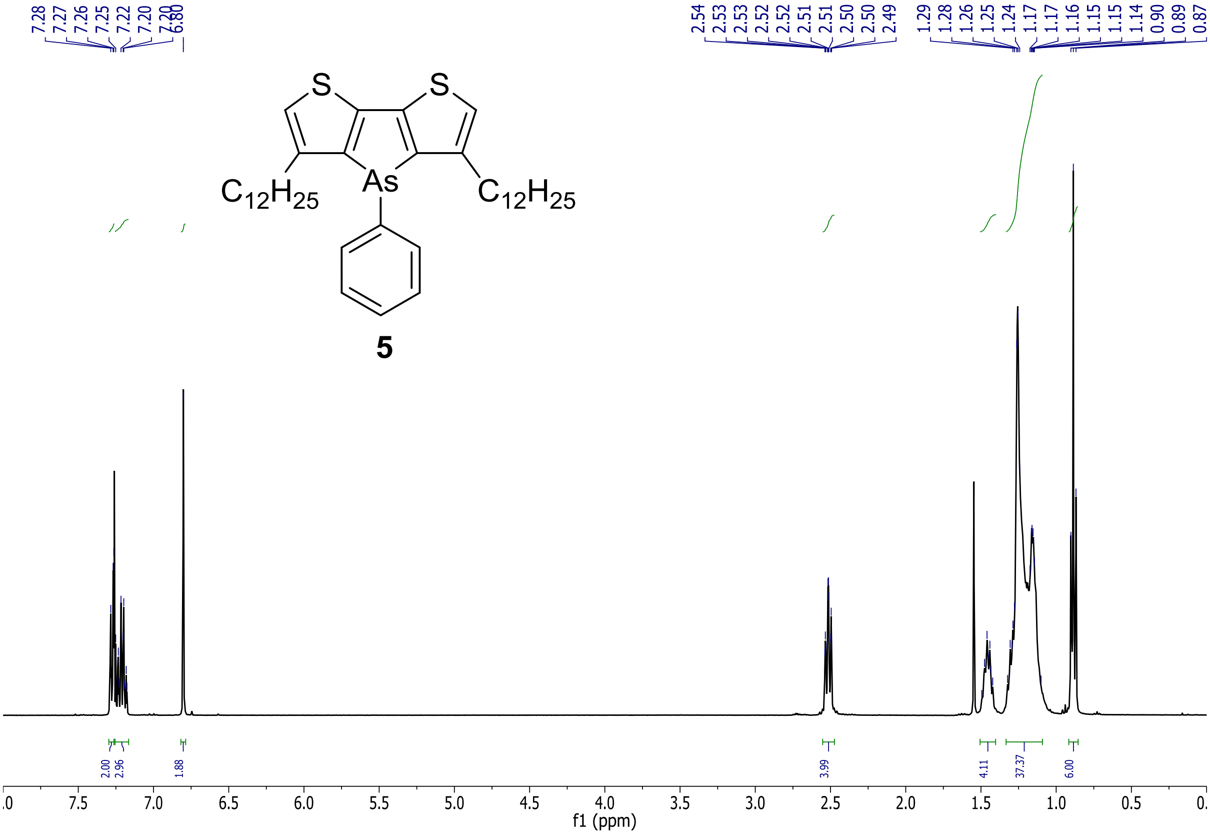

Figure S3: ^{1}H NMR spectrum of **5** (400 MHz, $CDCl_3$).

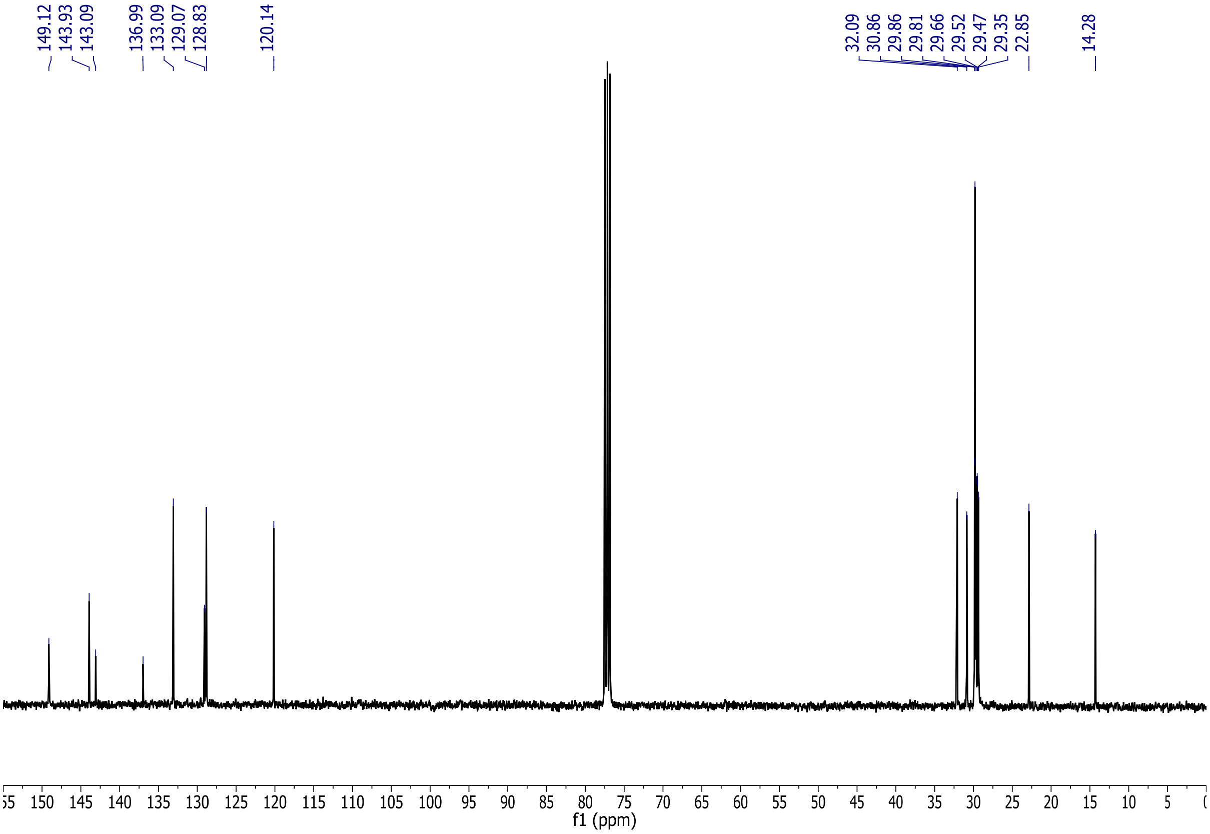

Figure S4: ^{13}C NMR spectrum of **5** (101 MHz, $CDCl_3$).

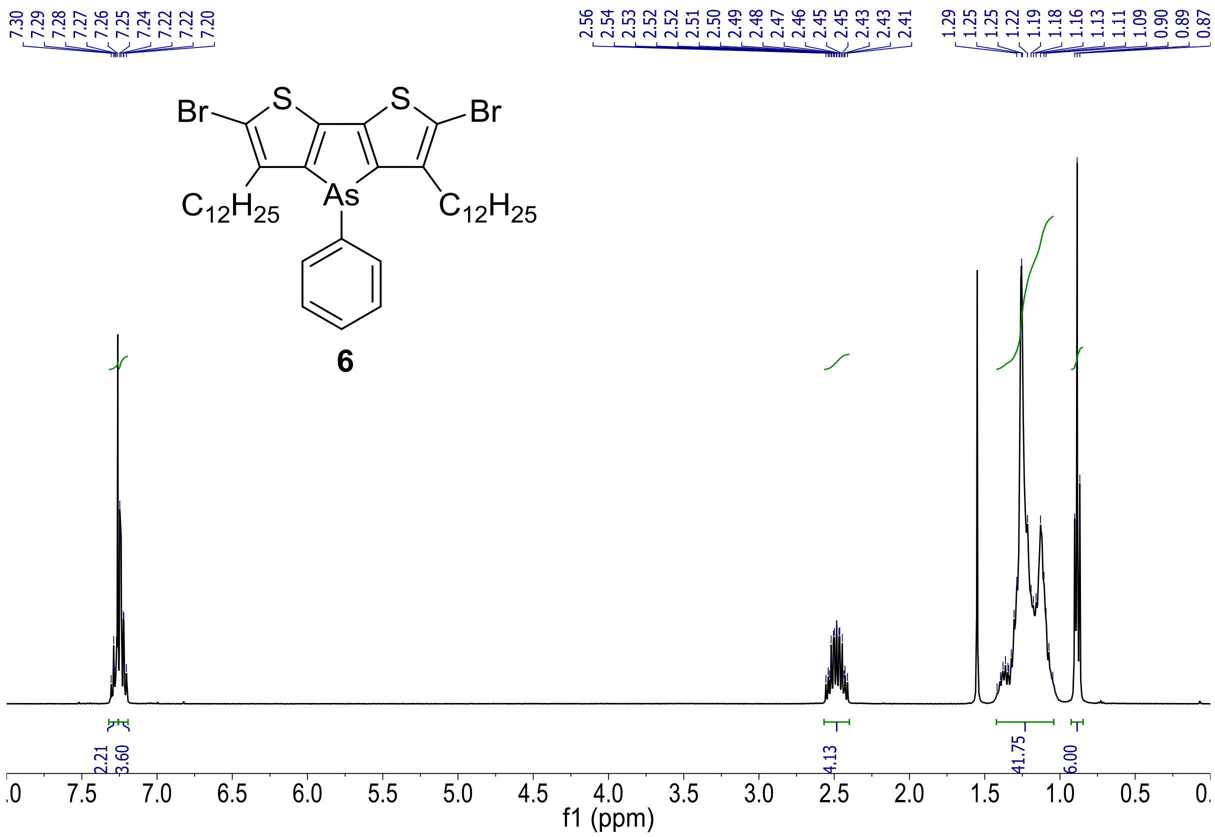

Figure S5: ^{1}H NMR spectrum of **6** (400 MHz, $CDCl_3$).

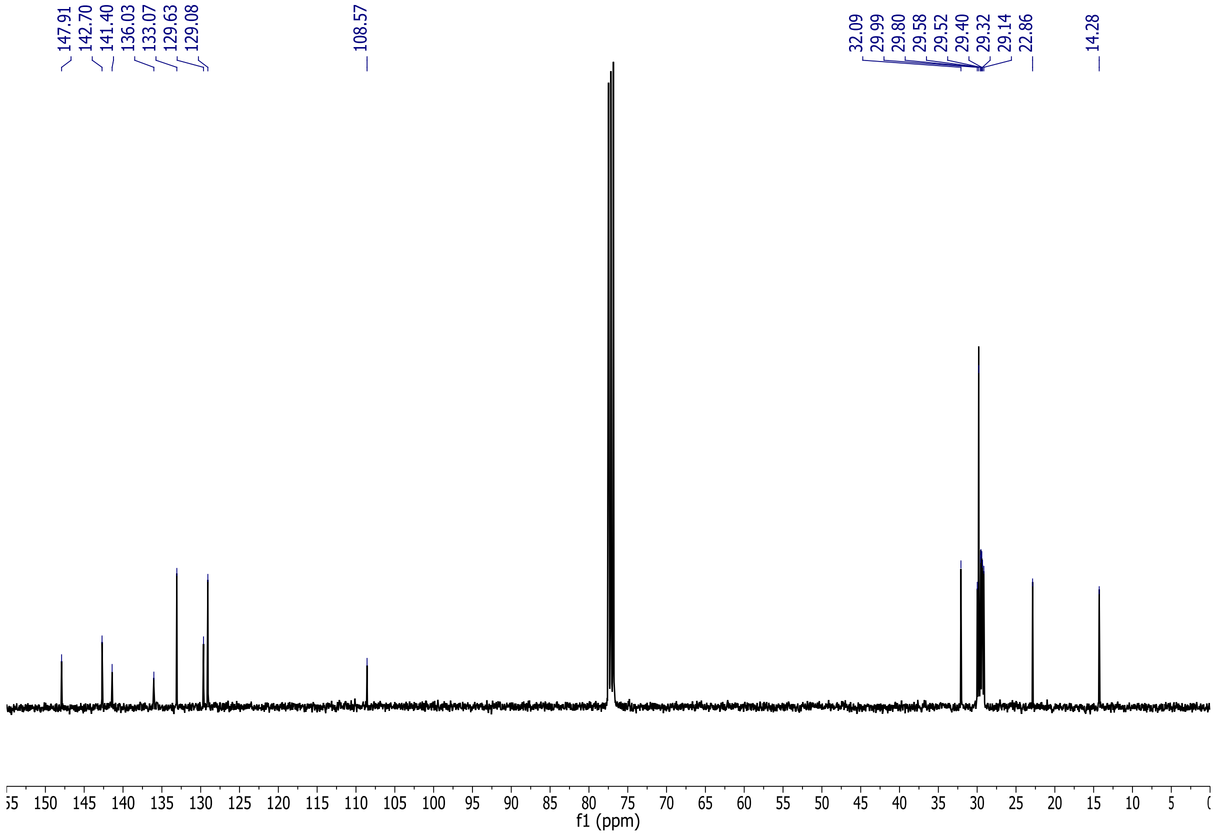

Figure S6: ^{13}C NMR spectrum of **6** (101 MHz, $CDCl_3$).

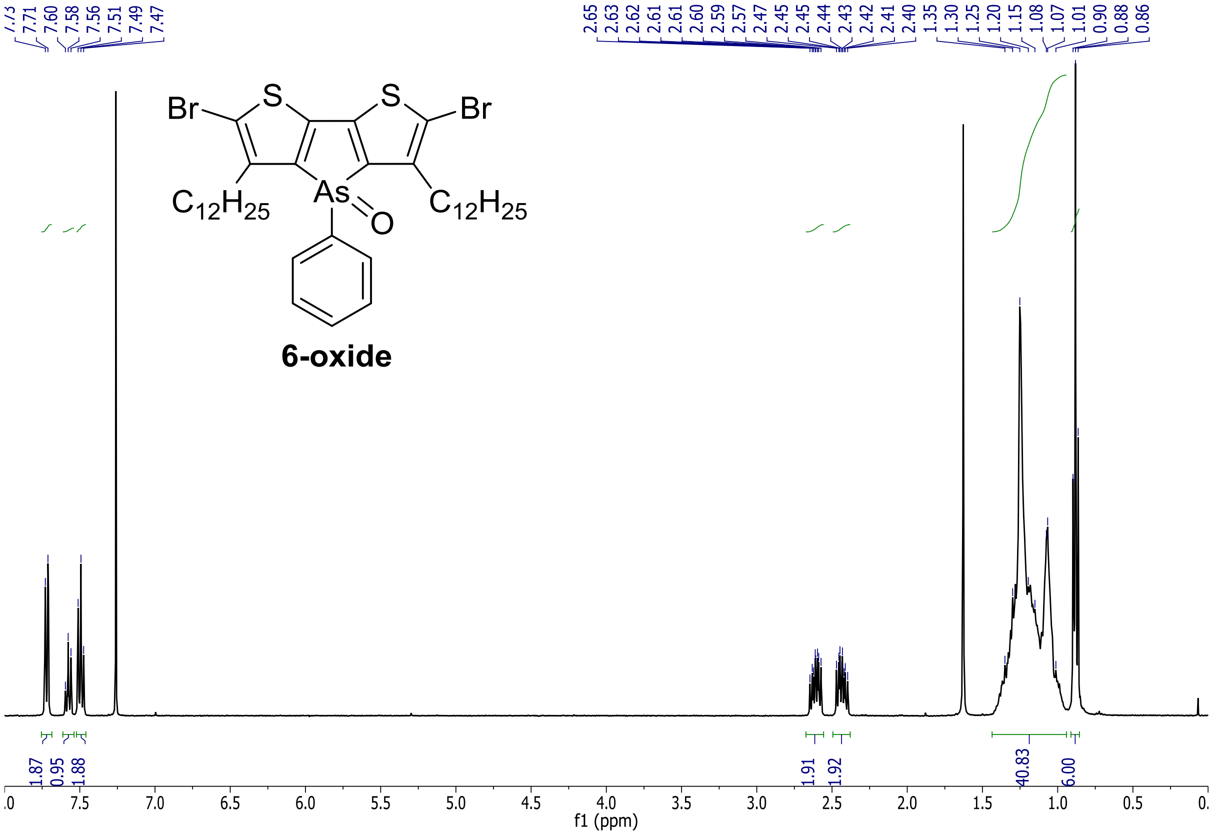

Figure S7: ^{1}H NMR spectrum of **6-oxide** (400 MHz, $CDCl_3$).

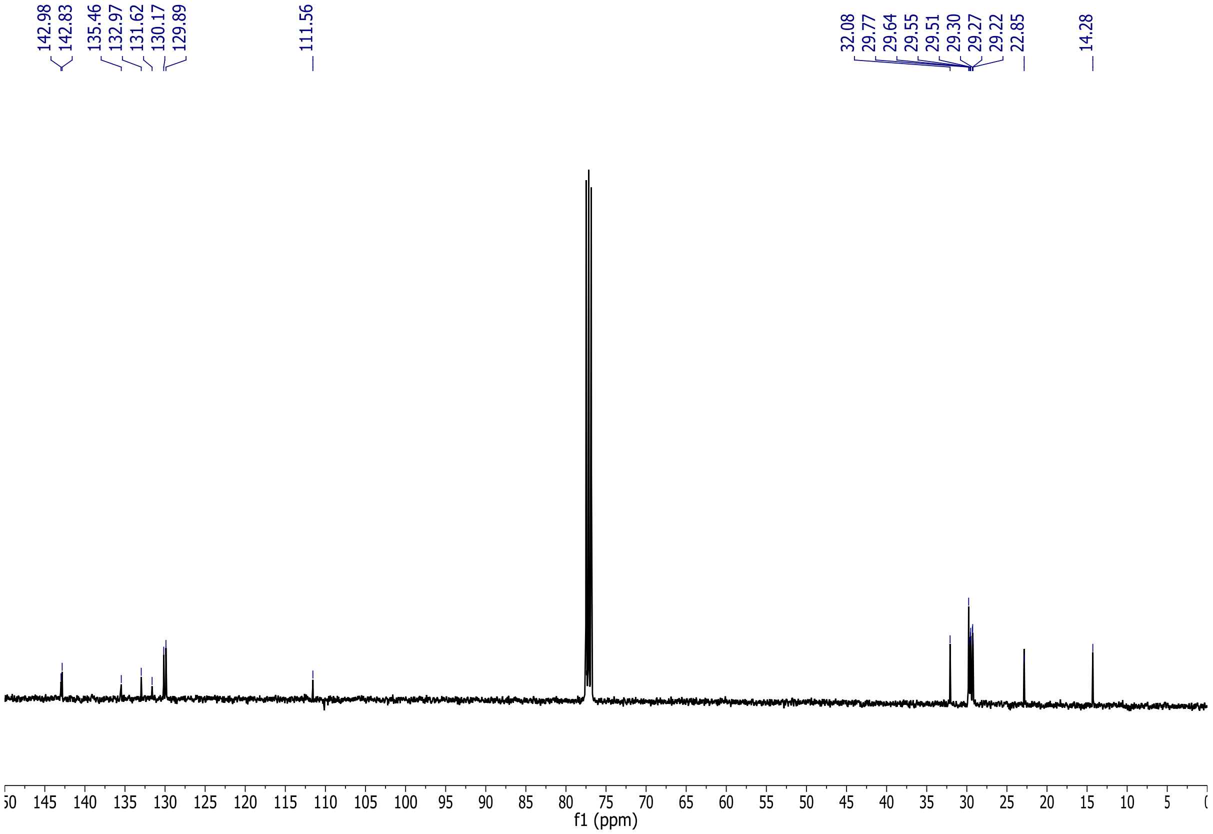

Figure S8: ^{13}C NMR spectrum of **6-oxide** (101 MHz, $CDCl_3$) .

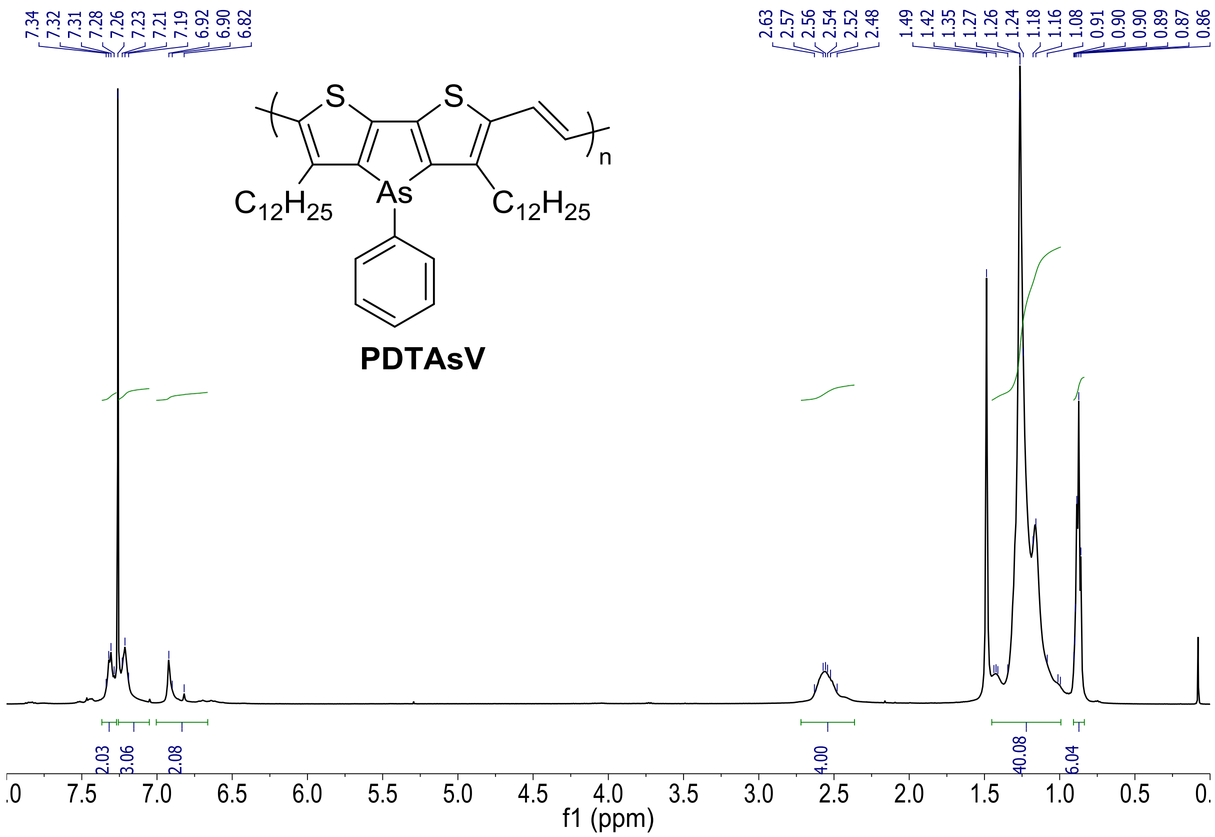

Figure S9: ^{1}H NMR spectrum of **PDTAsV** (500 MHz, 323 K, $CDCl_3$).

3. Heated UV of PDTAsV

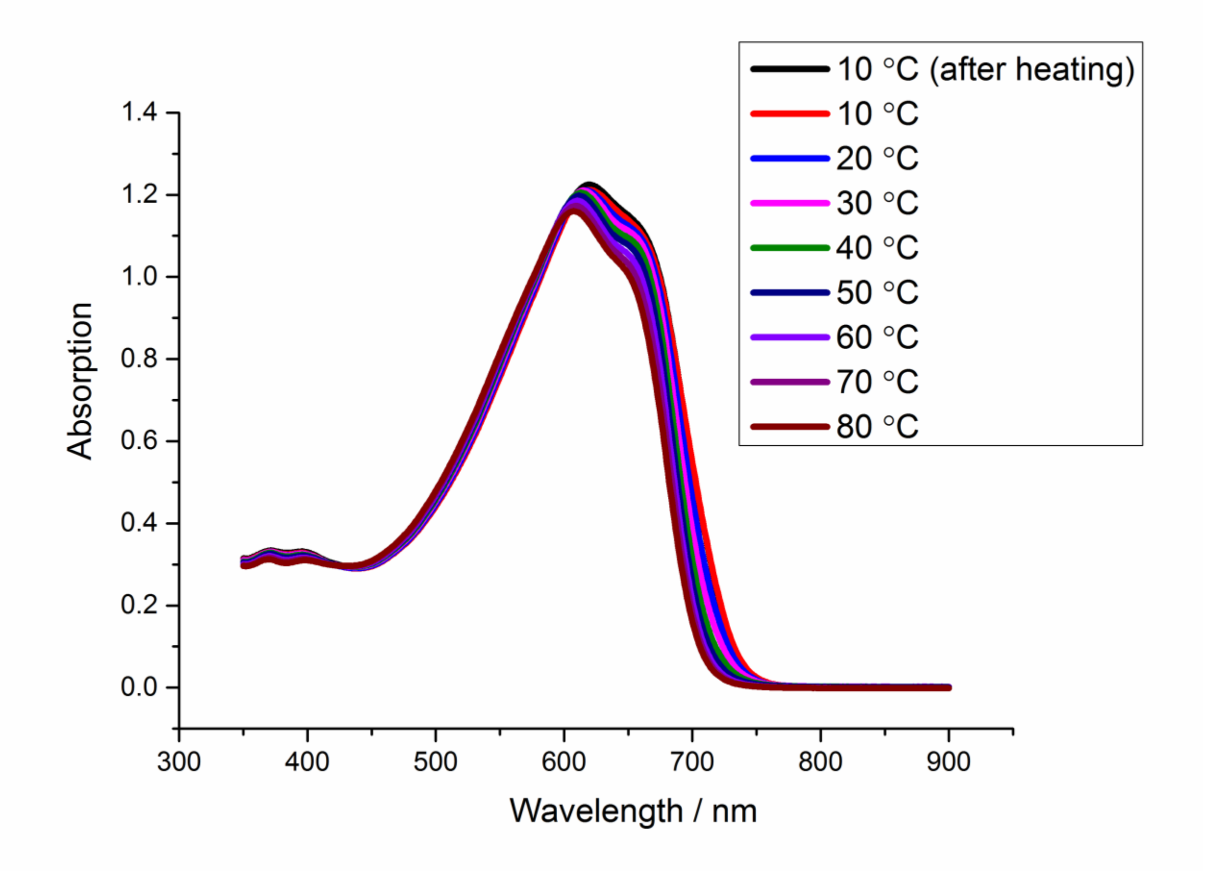

Figure S10: UV-vis absorption spectra of PDTAsV in chlorobenzene solution at different temperatures.

4. Frontier molecular orbitals of DTAs

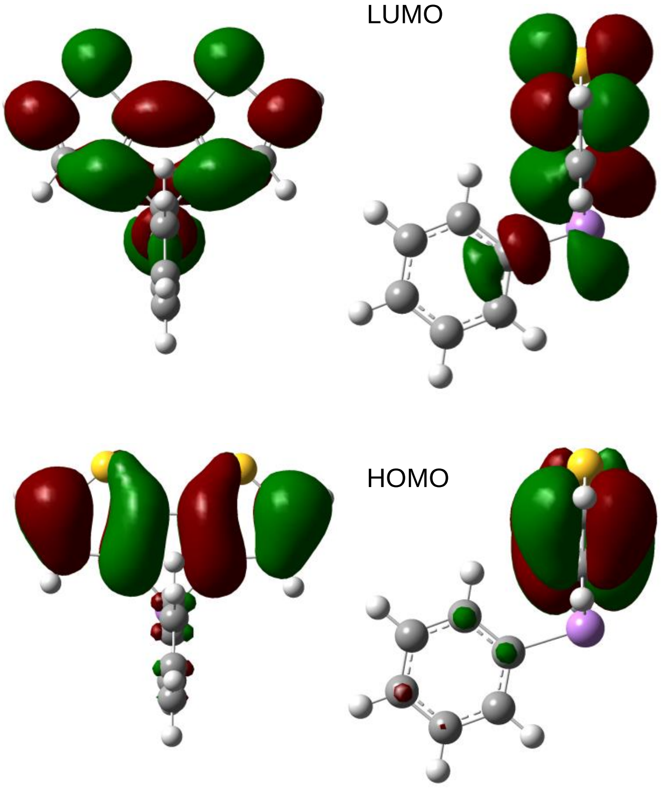

HOMOs are shown below LUMOs.

Figure S11: Frontier molecular orbitals of 3,5-didodecyl-4-phenyldithieno[3,2-b;2',3'-d]arsole (5).

5. OFET output characteristics

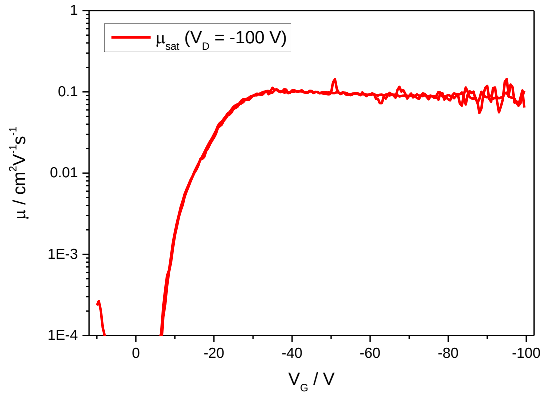

Figure S12: Mobility versus gate voltage based on first derivative of the square root of the saturation regime transfer curve.

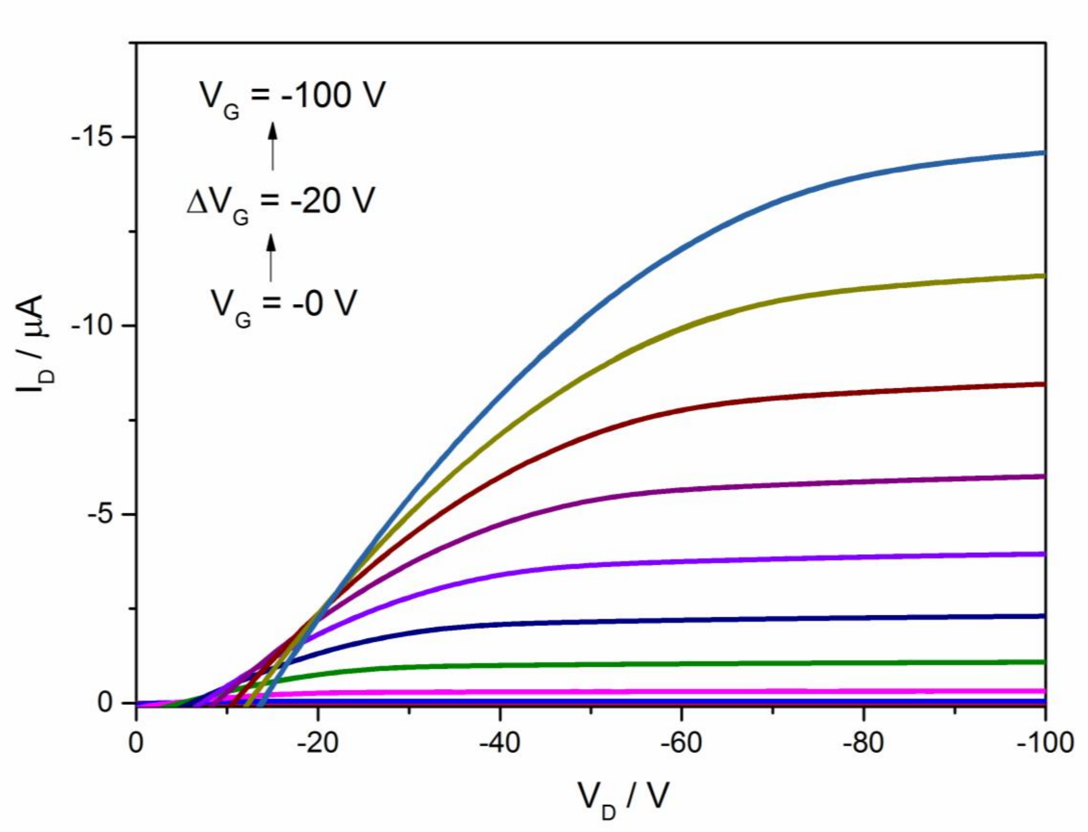

Figure S13: OFET output characteristics of a top gate, bottom contact device using PDTAsV as the active layer.

7. References

[1] Z. Fei, Y. Kim, J. Smith, E. B. Domingo, N. Stingelin, M. A. McLachlan, K. Song, T. D. Anthopoulos, M. Heeney, *Macromolecules* **2012**, *45*, 735.

[2] M. J. Frisch , G. W. Trucks , H. B. Schlegel , G. E. Scuseria , M. A. Robb , J. R. Cheeseman , G. Scalmani , V. Barone , B. Mennucci , G. A. Petersson , H. Nakatsuji , M. Caricato , X. Li , H. P. Hratchian , A. F. Izmaylov , J. Bloino , G. Zheng , J. L. Sonnenberg , M. Hada , M. Ehara , K. Toyota , R. Fukuda , J. Hasegawa , M. Ishida , T. Nakajima , Y. Honda , O. Kitao , H. Nakai , T. Vreven , J. A. Montgomery Jr. , J. E. Peralta , F. Ogliaro , M. J. Bearpark , J. Heyd , E. N. Brothers , K. N. Kudin , V. N. Staroverov , R. Kobayashi , J. Normand , K. Raghavachari , A. P. Rendell , J. C. Burant , S. S. Iyengar , J. Tomasi , M. Cossi , N. Rega , N. J. Millam , M. Klene , J. E. Knox , J. B. Cross , V. Bakken , C. Adamo , J. Jaramillo , R. Gomperts , R. E. Stratmann , O. Yazyev , A. J. Austin , R. Cammi , C. Pomelli , J. W. Ochterski , R. L. Martin , K. Morokuma , V. G. Zakrzewski , G. A. Voth , P. Salvador , J. J. Dannenberg , S. Dapprich , A. D. Daniels , Ö. Farkas , J. B. Foresman , J. V. Ortiz , J. Cioslowski , D. J. Fox , Gaussian 09, Revision C.01 , Wallinford, CT, USA 2009 .

[3] R. Betz, M. M. Reichvilser, E. Schumi, C. Miller, P. Klüfers, *Zeitschrift für Anorg. und Allg. Chemie* **2009**, *635*, 1204.

[4] D. G. Patel, K. R. Graham, J. R. Reynolds, *J. Mater. Chem*. **2012**, 22, 3004
